# Effects of non-genetically and genetically modified organism (maize-soybean) diet on growth performance, nutrient digestibility, carcass weight, and meat quality of broiler chicken

**DOI:** 10.5713/ajas.18.0723

**Published:** 2019-01-02

**Authors:** Song Zhang, Xiang Ao, In Ho Kim

**Affiliations:** 1 Department of Animal Resource and Science, Dankook University, Cheonan 31116, Korea; 2 Kemin Industries (Zhuhai) Co., Ltd. Sanzao, Zhuhai 519040, China

**Keywords:** Non-GMO Diet, GMO Diet, Growth Performance, Nutrient Digestibility, Broiler

## Abstract

**Objective:**

This study was conducted to compare growth performance, nutrient digestibility and meat quality of broilers fed a genetically modified organism (GMO) diet or a non-GMO diet.

**Methods:**

A total of 840 broilers with an initial body weight of 43.03 g per chick were randomly allocated into 1 of the following 2 dietary treatments lasted for 32 days (15 broilers per pen with 28 replicates per treatment): i) Trt 1, GMO maize-soybean meal based diet; ii) Trt 2, non-GMO maize soybean meal based diet. Both diets were maize-soybean meal diets. The GMO qualitative analysis, proximate analysis and amino acid analysis of the feed ingredient samples were carried out. Diets were formulated based on a nutrient matrix derived from analysis results. Growth performance was measured on day 0, 7, 17, and 32. And all other response criteria were measured on day 32.

**Results:**

The analysis results showed that the total Lys, Met, Thr of non-GMO grains were lower than that of GMO grains, the protein content of GMO soybean meal was higher than that of non-GMO soybean meal. Feed intake and feed conversion rate (FCR) were greater (p<0.05) in broilers provided with non-GMO diet than that of the GMO group from d 17 to 32. A decrease in FCR was observed in birds fed the GMO diet through the entire experiment (p<0.05). No significant impacts on blood profile, meat quality and nutrient digestibility were found in response to dietary treatments throughout the experimental period (p>0.05).

**Conclusion:**

These results indicated that non-GMO diet showed a negative effect on growth performance but nutrient digestibility, blood profile, carcass weight and meat quality were not affected by non-GMO diets.

## INTRODUCTION

In 2018, genetically modified organism (GMO) crops have been commercialized for 23 years. From 1996 to 2015, the cumulative area of transgenic crops reached 2 billion hectares worldwide. The first experiment on feeds with a genetically modified ingredient was published by Hammond et al [1]. Even though the United Nations, World Health Organization (WHO), Food and Agriculture Organization (FAO), the US Food and Drug Administration (FDA) and Environmental Protection Agency (EPA) have all stated that DNA, including DNA from transgenic crops, is a safe, natural component of food [2–6], concerns of the safety of genetically modified grains have been continuous. The public is concerned with the outcomes of technical risk assessments. They are also troubled about the uncertainty related to these outcomes, suspecting that risk assessments are based on an insufficient level of scientific knowledge [7,8]. Consequently, the risk assessments currently conducted especially may be not able to address long term effects of genetically modified foods. Ethical concerns are also important, for example, that a particular technology is in some way “tampering with nature” or that unintended effects are unpredictable and thus unknown to science [9].

In a democratic society where choice exists, people have rights to consume food that they believe to be safe. Since there is a certain need for non-GMO food including non-GMO animal protein, we need effective systems to assess non-GMO in feedstuffs from the nutritional point of view. In 2004, an animal feeding trial demonstrated that N7070bt maize diets supported broiler growth with mortality and feed conversion rate (FCR) similar to that supported by the N7070 isoline control [10]. In the same year, Kan et al [11] reported the GMO soybean containing gene bt-Cry1Ac protein was nutritionally equivalent to non-GMO soybean varieties when fed to broilers. However, with the rapid development of breeding and biomolecular technology, it is difficult to avoid GMO ingredients in animal nutrition research. When our experts determined databases of raw materials or recommendations of animal nutritional needs, the GMO and non-GMO ingredients were not considered respectively. Recently, our nutritionists may be better at using GMO-ingredient to formulate diet than non-GMO. Or, it seems easier for animal nutritionists to make use of GMO diets comparing to non-GMO diets. During the past 10 years no experiments were conducted to determine the effects of an absolute non-GMO diet on animals. Consequently, the objective of the study is to compare growth performance, nutrient digestibility and meat quality of broiler fed either a GMO diet or a non-GMO diet.

## MATERIALS AND METHODS

### Test and control corn and soybean meal

GMO and non-GMO maize and soybean samples were sent to independent laboratory Kogenebiotech Co., LTD (Seoul, Korea) for GMO analysis. GMO qualitative analysis of maize was performed by polymerase chain reaction (PCR) with the specific primer pairs for SSIIb (reference gene), 35S Promoter, NOS Terminator, DP-098140-6, and DAS-40278-9 genes respectively. GMO qualitative analysis of soybean was performed by PCR with the specific primer pairs for Lectin (reference gene), 35S Promoter, NOS Terminator, MON89788, DP305423-1, DO356043-5, MON87701, CV127, MON87708, MON87769, and DAS-68416-4 genes respectively. The results are shown in Table 1 and 2. The results confirmed that the maize and soybean meal were non-GMO.

### Corn and soybean meal analysis

Samples of each of the four lots of ingredients were used to carry out proximate analysis [12]. All raw materials formulated (non-GMO and GMO maize and soybean) in diet were provided by Daehan feed mill company which were imported from USA. Amino acid contents were determined, following acid hydrolysis with 6 N HCl at 110°C for 24 h, using an amino acid analyzer (Biochrom 20, Pharmacia Biotech, Cambridge, England) (Table 3).

### Animal, diet, experimental design

A total of 840 male Ross 308 two-day-old (body weight [BW] of 43.03±5 g) broiler chicks were obtained from a commercial hatchery (Yang Ji Company, Cheonan, Korea). All birds were randomly assigned into 2 dietary treatment groups by BW in a randomized complete block design. Each treatment had 28 replicate pens of 15 broilers in each pen. i) Trt 1, GMO maize-soybean meal based diet; ii) Trt 2, non-GMO maize soybean meal based diet. Both diets were maize-soybean meal diets. All birds were kept in stainless steel pens of identical size (1.75× 1.55 m) in a house with concrete floors covered with clean rice bran in an area that was provided with continuous light and were given free access to water and mash feed. During the experiment, house’s temperature was regulated around 32°C. All diets were formulated to contain approximately equal amounts of the 3 first limiting dietary essential amino acids (methionine, cystine, and lysine), Ca, absorbable P, and Na, based on the analytical data from the feedstuffs. All diets were formulated to meet or exceed the NRC [13] requirements for broilers. Chromium contents were 330 and 320 μg/kg in starter and finisher diets, respectively, as measured by atomic absorption spectrophotometer (Analyst 100, Perkin-Elmer, Norwalk, CT, USA). The initial and final diet compositions are shown in Table 4. All birds used in this trial were handled in accordance with the guidelines set forth by the Animal Care and Use Committee of Dankook University.

### Sampling and measurements

The broilers were weighed by pen and feed intake (FI) was recorded on d 0, 7, 17, and 32. This information was then used to calculate body weight gain, and FCR. For deaths during the middle of a weighing period, the dead animal’s weight was recorded, and the gain of the dead bird was counted towards pen gain in figuring feed conversion. Number of dead birds was examined as well. At the end of the experiment, 56 broilers were randomly selected from each treatment (2 birds per pen) and blood samples were collected in 5 mL vacuum tubes (Becton Dickinson Vacutainer System, Franklin Lakes, NJ, USA) and then centrifuged (3,000×g, 15 min, 4°C) within one hour after the collection of the sample to separate the serum. The blood urea nitrogen (BUN), creatinine, and glucose in the serum samples were analyzed with an automatic biochemical analyzer (HITACHI 747, Tokyo, Japan) using colorimetric methods.

After blood collection, total 56 broilers were weighed individually and slaughtered by cervical dislocation. The stomach, breast meat, bursa of Fabricius (*Bursa cloacalis*), liver, spleen, and abdominal fat were then removed by trained personnel and weighed. The breast muscles were stored at −20°C for the following analysis. Organ weight was expressed as a percentage of BW. The breast muscle Hunter L* (lightness), a* (redness), and b* (yellowness) values were determined using a Minolta CR410 chromameter (Konica Minolta Sensing Inc., Osaka, Japan). Cooking loss was determined using 5 g of breast meat, which was heat-treated in plastic bags separately in a water bath (100°C) for 5 min. Samples were cooled at room temperature. Cooking loss was calculated as (sample weight) before cooking – sample weight after cooking)/sample weight before cooking×100. A piece of breast meat was chilled at 2°C for 26 h, duplicate pH values for each sample were measured using a pH meter (Fisher Scientific, Pittsburgh, PA, USA).

### Statistical analysis

All data were subjected to the statistical analysis as a randomized complete block design using the general linear model procedures of SAS [14], and the cage was used as the experimental unit. Differences among treatment means were determined using the Duncan’s multiple range test. Statements of statistical significance were based on p<0.05.

## RESULTS AND DISCUSSION

### Test and control on corn and soybean meal

Only maize and soybean meal were included in the formula as the main raw materials to ensure that the non-GMO diets were applied in this experiment. The GMO qualitative analysis results are presented in Table 1 and 2. Four genes including 35S Promoter, NOS Terminator, DP-098140-6, DAS40278-9 were not detected non-GMO maize. 35S Promoter, NOS Terminator, MON89788, DP305423-1, DO356043-5, MON87701, CV127, MON87708, MON87769, DAS-68416-4 were not detected in non-GMO soybean meal. Those results confirmed that the maize and soybean meal which applied in non-GMO meal were non-GMOs.

### Corn and soybean meal analysis

The results of proximate analysis and amino acid analysis presented in Table 3 were reported as the percentage by weight on an as-is basis. In maize, the crude protein of non-GMO maize was 0.17% higher than that of GMO maize. Nevertheless, the higher content of Lys, Met, Thr was found in GMO maize. In soybean meal, the crude fiber of non-GMO soybean was 3.02% higher than that of GMO soymeal meal. A higher content of crude protein was observed in GMO soybean meal as well. Besides, the contents of Lys and Met in GMO soybean were 0.6% and 0.8% higher than that in non-GMO soybean, respectively. The development of GMO crops, especially the first generation, enhance insect or herbicide resistance, abiotic stress tolerance. The nutrient compositions of GMO grain are better than that of non-GMO materials. Probably due to fewer challenging factors (insect, herbicide or abiotic stress) affecting the accumulation of nutrients in the growth process. Besides, Rayan et al [15] reported that there were some statistical differences between the GMO corn samples and non-GMO control in some biochemical components. But he believed that those results were unlikely to be biologically significant, since they were well within the range of literature values.

### Growth performance and nutrient digestibility

The results of growth performance and nutrient digestibility are presented in Table 5 and 6. The FI and FCR were greater (p<0.05) in broilers provided with non-GMO diet feed than that in the GMO group from day 17 to 32. A decrease FCR was observed when birds were fed with the GMO diet through the whole experiment (p<0.05). In 1997, A program was started to assess GMO including Bt-maize, Pat-maize, Pat-sugar beets and Gt-soybeans, which tried to determine an effective system to assess GMO in feed stuffs from the view of nutrition. In 2001, the series of experiments reported by Flachowsky et al [16] had been published by Aulrich et al [17,18], Bohme et al [19], Daenicke et al [20,21], and Halle et al [22]. Results of all the experiments did not show any significant difference in growth performance and nutrient digestibility between GMO diet and non-GMO diet. The latest article on non-GMO Feed stuff in broilers was published in 2010 by Świątkiewicz et al [23], which revealed that no statistical difference was observed in any of the performance parameters across dietary treatments. Only two articles showed some improvements in growth performances when birds were fed the GMO-diet, one of the two papers published in 1998 showed the birds receiving GMO-corn diets exhibited improved adjusted feed conversion ratios at 28 and 38 days of age. The author considered that the improved feed conversion ratios cannot necessarily be attributed to the corn source, but these data did show an absence of any deleterious effects associated with the diets made from GMO when compared to diets made from non-GMO corn [24]. However, our study found that the GMO-diet exhibited a better growth performance of FCR than non-GMO diet. Chicks fed non-GMO diet have higher FCR than those fed GMO diet which was attributed to the higher food intakes. The first reason for these differences may be a higher apparent metabolic energy value assumed for diet formulation. The metabolic energy (ME) value of the experimental diets are calculated based on CVB Table Booklet Feeding of poultry recommendation [25]. It is not clear whether standard non-GMO maize or soybean meal supplied the similar ME as much as GMO maize for diet. During age of d 17 to d 32, broilers are in faster growth than that at age of 2 weeks. Goliomytis [26] documented the growth rate from 3 weeks to 6 weeks of BW, breast weight and leg weight were greater than that in first 2 weeks in broilers. Therefore, the protein and amino acids could be crucial one of all elements. The second reason may be different amino acids compositions or protein characteristics between GMO and non-GMO diet. The lower content of first 3 limiting essential amino acids occur in non-GMO feed stuff, the more crystalline amino acids have to be used in a non-GMO diet. But, the nutritional value of protein in animal feed may be related not only to amino acid content and composition, but also to the different resource of protein characteristics. Differences in physical and chemical properties of protein resource also affect the release dynamics of amino acids in the digestive tract of animals. The GMO varieties of plants which were called second-generation crops have traits to enhance nutritional properties [27]. Furthermore, the branched-chain amino acids which plays several critical roles in metabolism homeostasis and cell functions including immunity, survival and growth, energy homeostasis, and protein and lipid metabolism regulation [28] were not calculated in diet. Our amino acid analysis showed the quantity of branched-chain amino acids in non-GMO grain were around 1.15 times as much as that in non-GMO grain. Therefore, the relatively lower content of branched-chain amino acids in non-GMO diets might be another reason for the influence.

### Blood profile, meat quality, and organ weight

No significant impact on blood profile, meat quality, organ weight was found in response to the two treatments throughout the experimental period (Tables 7, 8) (p>0.05). Serum creatinine (a blood measurement) is an important indicator of renal health because it is an easily-measured by-product of muscle metabolism that is excreted unchanged by the kidneys. Plasma or BUN concentration may be useful as an indicator of protein status within a group of animals as well as nitrogen utilization and could help to fine-tune diets or identify problems with a feeding program [29]. Those results might demonstrate that the metabolism of protein and amino acids are not affected in the birds fed non-GMO and GMO diets. Consistent with our research, almost all previous published papers demonstrated that no statistic difference in carcass and organ weight was found between GMO and non-GMO feed. For example, the study of Tatlor et al [30] showed that no difference in carcass characteristics was present between birds were fed either GMO-maize or non-GMO maize.

## CONCLUSION

In conclusion, a non-GMO maize-soybean basal diet had no adverse effects on blood profile, carcass characteristic and meat quality in broilers. However, growth performance was reduced when the birds were fed a non-GMO diet. Further experiments are required to determine the reason for this result.

## Figures and Tables

**Table 1:** Qualitative analysis of genetically modified maize

Analysis item1)	Non-maize
SSIIb	Detected
35S Promoter	Not detected
NOS Terminator	Not detected
DP-098140-6	Not detected
DAS40278-9	Not detected

1) Independent laboratory Kogenebiotech Co. LTD reported.

**Table 2:** Qualitative analysis of genetically modified soybean meal

Analysis item1)	Non-soybean meal
Soybean reference gene (lectin)	Detected
35S Promoter	Not detected
NOS Terminator	Not detected
MON89788	Not detected
DP305423-1	Not detected
DO356043-5	Not detected
MON87701	Not detected
CV127	Not detected
MON87708	Not detected
MON87769	Not detected
DAS-68416-4	Not detected

1) Independent laboratory Kogenebiotech Co. LTD reported.

**Table 3:** Compositions of corn and soybean meal sample

Analyses1)	Maize		Soybean meal	
	
	Non-GMO	GMO	Non-GMO	GMO
Proximate analyses (%)				
Moisture	12.3	12.1	11.5	11.6
Crude fat	3.26	3.82	1.37	1.72
Crude protein	8.03	7.86	45.89	46.3
Crude fibre	2.43	1.67	6.53	3.51
Amino acids (%)				
Lys	0.21	0.23	2.75	2.81
Met	0.14	0.16	0.51	0.59
Cys	0.15	0.17	0.65	0.68
Thr	0.27	0.27	1.79	1.84
Val	0.31	0.36	2.06	2.10
Ile	0.21	0.26	2.01	1.98
Leu	0.84	0.91	3.46	3.43
Phe	0.36	0.36	2.30	2.28
His	0.22	0.23	1.28	1.26
Arg	0.33	0.36	3.32	3.23
Pro	0.54	0.68	1.76	1.93
Asp	0.48	0.50	5.48	5.38
Ser	0.37	0.36	2.30	2.35
Glu	1.33	1.36	8.27	8.30
Gly	0.28	0.29	2.00	1.94
Ala	0.51	0.56	1.85	1.95

1) Reported on an as-is basis.

**Table 4:** Basal diet composition (as-fed basis)

Items	Starter1)		Grower1)		Finisher1)	
		
	TRT12)	TRT22)	TRT12)	TRT22)	TRT12)	TRT22)
Ingredient (%)						
Maize (GMO)	57.41	-	60.29	-	62.63	-
SBM (GMO)	36.65	-	33.03	-	29.56	-
Maize (Non-GMO)	-	56.81	-	59.67	-	62.37
SBM (Non-GMO)	-	36.70	-	33.01	-	29.46
Tallow	1.54	1.95	2.50	2.83	3.56	3.79
Limestone	1.70	1.75	1.62	1.66	1.74	1.77
MDCP	1.39	1.37	1.32	1.31	1.36	1.35
Salt	0.31	0.31	0.31	0.31	0.31	0.31
DL-methionine (99%)	0.36	0.42	0.32	0.38	0.36	0.42
L-lysine-HCl (98.5%)	0.23	0.26	0.22	0.25	0.14	0.17
L-threonine (98.5%)	0.11	0.13	0.09	0.11	0.04	0.06
Choline (60%)	0.10	0.10	0.10	0.10	0.10	0.10
Vitamin premix3)	0.10	0.10	0.10	0.10	0.10	0.10
Mineral premix4)	0.10	0.10	0.10	0.10	0.10	0.10
Total	100.0	100.0	100.0	100.0	100.0	100.0
Calculated composition (%)						
Crude protein	22.00	22.00	20.50	20.50	19.00	19.00
Crude fiber	2.24	3.77	2.16	3.61	2.09	3.44
Crude fat	4.35	4.29	5.35	5.21	6.45	6.21
Ash	6.23	6.35	5.91	6.00	5.78	5.94
ME (kcal/kg)	3,000	3,000	3,100	3,100	3,200	3,200
Ca	0.95	0.95	0.90	0.90	0.90	0.90
AP	0.4	0.4	0.38	0.38	0.49	0.50
SID-Lys	1.25	1.25	1.15	1.15	1.00	1.00
SID-TSAA	0.95	0.95	0.87	0.87	0.76	0.76
SID-Thr	0.85	0.85	0.78	0.78	0.68	0.68

GMO, genetically modified organism; SBM, soybean meal; MDCP, mono-calcium and di-calcium phosphate; ME, metabolic energy; AP, available phosphorus; SID, standard ileal digestibility; TSAA, total sulfur amino acid.

1) Starter diets, provided during weeks 0 to 1; grower diets, provided during weeks 2 to 5.

2) TRT1, GMO corn-SBM diet; TRT2, non-GMO corn-SBM diet. Each treatment had 28 replicate pens of 15 broilers in each pen.

3) Provided per kg of diet: 15,000 IU of vitamin A, 3,750 IU of vitamin D_3_, 37.5 mg of vitamin E, 2.55 mg of vitamin K_3_, 3 mg of thiamin, 7.5 mg of riboflavin, 4.5 mg of vitamin B_6_, 24 μg of vitamin B_12_, 51 mg of niacin, 1.5 mg of folic acid, 0.2 mg of biotin and 13.5 mg of pantothenic acid.

4) Provided per kg of diet: 37.5 mg Zn (as ZnSO_4_), 37.5 mg of Mn (MnO_2_), 37.5 mg of Fe (as FeSO_4_·7H_2_O), 3.75 mg of Cu (as CuSO_4_·5H_2_O), 0.83 mg of I (as KI), and 0.23 mg of Se (as Na_2_SeO_3_·5H_2_O).

**Table 5:** Effect of GMO and non-GMO corn-SBM diets on growth performance of broilers

Items	TRT11)	TRT21)	SEM
Initial BW (g)	43	43	0
d 7 BW	135	133	1
d 17 BW	573	576	5
d 32 BW	1,706	1,679	14
d 1–7			
BWG (g)	92	90	1
FI (g)	106	108	1
FCR	1.154	1.196	0.015
d 7–17			
BWG (g)	438	429	5
FI (g)	611	595	6
FCR	1.398	1.390	0.129
d 17–32			
BWG (g)	1,134	1,117	14
FI (g)	1,724^b^	1,762^a^	11
FCR	1.524^b^	1.579^a^	0.014
Overall			
BWG (g)	1,663	1,636	13
FI (g)	2,440	2,464	12
FCR	1.468^b^	1.507^a^	0.009
Mortality rate (%)	1.743	1.966	0.36

GMO, genetically modified organism; SBM, soybean meal; SEM, standard error of means; BW, body weight; BWG, body weight; FI, feed intake; FCR, feed conversion rate.

1) TRT1, GMO corn-SBM diet; TRT2, non-GMO corn-SBM diet. Each treatment had 28 replicate pens of 15 broilers in each pen.

a,b Means in the same row with different superscripts differ (p<0.05).

**Table 6:** Effect of GMO and non-GMO corn-SBM diets on nutrient digestibility of broilers

Items (%)	TRT11)	TRT21)	SEM
Dry matter	73.92	74.13	0.69
Nitrogen	71.11	72.10	0.61

SEM, standard error of means; SBM, soybean meal.

1) TRT1, GMO-SBM diets; TRT2, non-GMO corn-SBM diets. Each treatment had 28 replicate pens of 15 broilers in each pen.

**Table 7:** Effect of GMO and non-GMO corn-SBM diets on blood profile of broilers

Items (mg/dL)	TRT11)	TRT21)	SEM
Blood urea nitrogen	2.75	2.25	0.46
Blood creatinine	0.16	0.18	0.01
Blood glucose	225.00	228.75	0.95

SEM, standard error of means; SBM, soybean meal.

1) TRT1, GMO corn-SBM diets; TRT2, non-GMO corn-SBM diets. Each treatment had 28 replicate pens of 15 broilers in each pen.

**Table 8:** Effect of GMO and non-GMO corn-SBM diets on meat quality and organ weigh of broilers

Items	TRT11)	TRT21)	SEM
pH value	5.55	5.50	0.06
Breast muscle color			
Lightness (L*)	53.38	53.97	0.66
Redness (a*)	10.28	10.29	0.42
Yellowness (b*)	9.13	9.14	0.23
Cooking loss	34.65	34.30	0.16
WHC (%)	51.32	52.74	1.69
Drip loss (%)			
d 1	2.87	2.75	0.20
d 3	5.58	5.51	0.05
d 5	8.69	8.69	0.03
d 7	10.78	10.70	0.13
Relative organ weight (%)			
Breast muscle	18.51	18.54	0.11
Liver	2.51	2.54	0.09
*Bursa cloacalis*	0.12	0.13	0.01
Abdominal fat	3.42	3.54	0.16
Spleen	0.10	0.11	0.01
Gizzard	1.13	1.12	0.01

SEM, standard error of means; WHC, water holding capacity; SBM, soybean meal.

1) TRT1, GMO corn-SBM diets; TRT2, non-GMO corn-SBM diets. Each treatment had 28 replicate pens of 15 broilers in each pen.

a,b Means in the same row with different superscripts differ (p<0.05).
